# Peripheral Blood Derived Mononuclear Cells Enhance the Migration and Chondrogenic Differentiation of Multipotent Mesenchymal Stromal Cells

**DOI:** 10.1155/2015/323454

**Published:** 2015-01-12

**Authors:** Niina Hopper, John Wardale, Daniel Howard, Roger Brooks, Neil Rushton, Frances Henson

**Affiliations:** ^1^Division of Trauma and Orthopaedic Surgery, University of Cambridge, Addenbrooke's Hospital, Hills Road, Cambridge BC2 0QQ, UK; ^2^Department of Veterinary Medicine, University of Cambridge, Madingley Road, Cambridge CB3 0ES, UK

## Abstract

A major challenge in cartilage repair is the lack of chondrogenic cells migrating from healthy tissue into damaged areas and strategies to promote this should be developed. The aim of this study was to evaluate the effect of peripheral blood derived mononuclear cell (PBMC) stimulation on mesenchymal stromal cells (MSCs) derived from the infrapatellar fat pad of human OA knee. Cell migration was measured using an xCELLigence electronic migration chamber system in combination with scratch assays. Gene expression was quantified with stem cell PCR arrays and validated using quantitative real-time PCR (rtPCR). In both migration assays PBMCs increased MSC migration by comparison to control. In scratch assay the wound closure was 55% higher after 3 hours in the PBMC stimulated test group (*P* = 0.002), migration rate was 9 times faster (*P* = 0.008), and total MSC migration was 25 times higher after 24 hours (*P* = 0.014). Analysis of MSCs by PCR array demonstrated that PBMCs induced the upregulation of genes associated with chondrogenic differentiation over 15-fold. In conclusion, PBMCs increase both MSC migration and differentiation suggesting that they are an ideal candidate for inclusion in regenerative medicine therapies aimed at cartilage repair.

## 1. Introduction

Articular cartilage has limited reparative abilities and purely chondral defects do not heal spontaneously. Progress of degenerated tissue of the surrounding cartilage may lead to osteoarthritis (OA) [1]. However, articular cartilage injuries that penetrate the subchondral bone can undergo spontaneous repair through the formation of fibrocartilage [2]. Shapiro and coworkers [3] showed that this repair is mediated by the proliferation and differentiation of mesenchymal stromal cells (MSCs) that invade the defect from the underlying bone marrow and vasculature. This physiological repair response still forms the rationale behind a number of orthopaedic procedures described as bone marrow stimulation techniques [4].

Steadman et al. [5] first described the microfracture procedure to enhance chondral resurfacing by introducing multiple holes below the articular cartilage defect into the exposed subchondral bone plate. The healing capability of this technique is based on the formation of a blood clot and migration of cells from the bone marrow. The functional long term outcome after microfracture surgery has presented variable results, the main shortcomings including limited hyaline repair tissue, variable repair cartilage volume, and possibility of progressive ossification of the regenerated tissue [6, 7].

Autologous cell implantation techniques have been developed [8] to overcome the degeneration of repair tissue. However, finding an abundant source of healthy cells for cell therapy remains a challenge [9–11]. The use of autologous chondrocytes for cartilage repair strategies in older subjects may be limited by both age-related and disease-associated decline in chondrogenesis [12]. Currently, work on finding viable alternatives to chondrocytes is increasing, particularly as research on stem cell potential continues to grow [13–16].

Although articular cartilage has poor repair ability, increased joint remodeling with the formation of chondroosteophytes and loose bodies are common in OA suggesting some potential for repair activity [17]. One possible explanation for this could be that other tissue sources of suitable cells are present within the joint. The reports by Jones et al. [18] and English et al. [17] have documented multipotential and chondrogenic MSCs in both OA synovial fluid (SF) and OA Hoffa's fad pad. Hoffa's infrapatellar fat pad is situated under and behind the patella within the knee and can be resected with minimal morbidity [19]. Infrapatellar fat pad has been shown to contain multipotential mesenchymal/stromal cells that can be easily isolated and expanded in large numbers retaining good chondrogenic capacity on extended passaging [15, 17, 19–22].

Previous studies have shown that MSCs are systemically or locally recruited to the site of injury in the early inflammatory phase [23–25]. Low and inefficient homing of MSCs is considered to be a major limitation of existing MSC-based therapeutic approaches [26–28]. The mechanism by which MSCs home to tissues and migrate across endothelium is not yet fully understood. However, many of the molecules known to be involved in the tethering, rolling, adhesion, and transmigration of leukocytes from the bloodstream into tissues are known to also be expressed on MSCs [29–31].

There is evidence that MSCs can respond to chemotactic signaling molecules such as SDF-1/CXCR4 pathways [24], monocyte chemotactic protein-3 (MCP-3) [30], and chemotactic factors secreted by OA synovium and cartilage [32]. Chemokines are small, chemoattractant cytokines that play a key role in the recruitment of leukocytes to sites of inflammation and injury [33]. Many proinflammatory cytokines are released from the injury site in the early inflammatory phase [33, 34]. Growth factors and cytokines circulate in the peripheral blood and reach articular cartilage through the synovial fluid and several have been proposed as potential chemoattractants in cartilage repair including FGF, PDGF, VEGF, IGF-1, IL-8, BMP-4, BMP-7, TGF-*β*, and SDF-1 [35, 36].

Peripheral blood cells are known to secrete circulating cytokines and therefore the authors hypothesized that peripheral blood derived mononuclear cells (PBMCs) might offer an easily available way to deliver these chemokines therapeutically. The main aim of this study was to investigate whether PBMCs can induce infrapatellar fat pad derived MSC migration. Secondly, this study set out to evaluate the effect of PBMC stimulation on MSC lineage commitment and chondrogenic potential.

## 2. Materials and Methods

### 2.1. Infrapatellar Fat Pad Derived Mesenchymal Stem Cells

Human tissue was obtained from patients undergoing total joint replacement with full ethical consent (06/Q0108/213). The size of the infrapatellar fat pad varied greatly between different patients. The infrapatellar fat pad was minced using a sterile scalpel blade and placed in media containing 10% heat inactivated FBS (fetal bovine serum), penicillin/streptomycin (100 international units/mL and 100 *μ*g/mL), gentamycin (10 ng/mL), and amphotericin B (2.5 *μ*g/mL). The average age of seven consecutive donors was 71.4 ± 8.3 years with four female and three male donors. The individual tissue samples used in this study were obtained from separate donors at different times and hence processed at different times.

The cells were released from the tissue by digesting with collagenase A (11088793001, Roche) 0.2% w/v in complete media for three hours on an orbital shaker at 37°C. Once digested, the cell suspension was strained through 100 *μ*m cell strainers (Falcon) and centrifuged (400 g for 5 minutes) to obtain a cell pellet. This pellet was washed twice in media and the cells counted with a scepter 2.0 (Millipore) handheld automated cell counter. For standard monolayer cultures, the cells were plated on tissue culture plastic at a density of 20,000 cells/cm^2^ and cell passages 3–5 were used in the experiments.

### 2.2. Blood Derived Mononuclear Cells

Peripheral blood samples were taken from healthy volunteers with full ethical consent using a sterile Monovette Starsted EDTA 9 mL tube. Peripheral blood mononuclear cell suspension was prepared from fresh whole blood of 12 young (32.9 ± 9.3) healthy volunteers (4 female and 8 male donors). Briefly, the blood was diluted 1 : 1 with HBSS (without calcium or magnesium), gently layered on LymphoPrep solution, and centrifuged for 20 minutes at 800 g. The mononuclear cell-rich band was removed and resuspended in medium supplemented with 10% FBS and cells pelleted by centrifugation for 10 minutes at 250 g [37].

### 2.3. Microenvironment

Standard cell culture conditions comprised a humidified atmosphere of 5% CO_2_ in air inside an incubator. In order to mimic the physiological oxygen tension of hypoxic tissues, an environment comprising 90% nitrogen, 5% oxygen, and 5% CO_2_ was achieved using a hypoxia chamber and controller unit (ProOx model C21, BioSpherix, NY, USA) situated in a cell culture incubator. Unless otherwise stated, the cultures were under normal atmospheric oxygen tension (~20%). All reagents used for cell culture were purchased from Invitrogen unless otherwise specified.

### 2.4. Cell Phenotyping

Flow cytometry was used to characterize CD90 and CD105 cell surface markers using CD90-FITC Mouse IgG (IM1839U) and CD105-PE Mouse IgG (A07414, Beckman Coulter). Additionally, CD34 (IM1870) and CD45 (A07782, Beckman Coulter) were used for negative selection. Fluorochrome-conjugated antibodies were incubated in 20 *μ*L/2.0 × 10^5^ cell suspension in full media or PBS for 20 minutes at room temperature protected from light. Finally the cells were washed three times with PBS solution, resuspended into IsoFlow Sheath Fluid (8546859, Beckman Coulter), and the cell fluorescence was measured using Beckman Coulter Cytomics FC500 instrument. The data was assessed with Kaluza analysis software. Positivity for each antibody was defined as the level of fluorescence >99% of the isotype-matched control antibodies.

### 2.5. Multilineage Analysis

Multilineage differentiation was induced in this study with differentiation medium. MSCs were cultured as a monolayer with 1.5 × 10^5^ cells per well on a 12-well plate for 21 days. After confluency, the cells were treated with three differentiation media in triplicates with basic media used as a negative control. At the end of the experiment, the cells were fixed, stained, and analyzed under a light microscopy.

For osteogenic differentiation [38–40] the medium consisted of 50 *μ*g/mL L-ascorbic acid 2-phosphate (A8960-5G, Sigma), 10 mM *β*-glycerol phosphate (G9422-10G, Sigma), and 10 nM dexamethasone (50-02-2, Sigma). The medium was changed every 3-4 days for over a period of 21 days. At the end of the experiment, the osteogenic cultures were fixed in 70% ethanol on ice for 1 hour and then stained with 2.0% alizarin red solution for 10 minutes at room temperature and finally washed three times with PBS.

To promote chondrogenic differentiation, StemPro Chondrogenesis Supplement (A10064-10, Gibco, Paisley, UK) was added to the basal medium and the medium was changed every 3-4 days for over a period of 21 days [41]. Chondrogenic cultures were fixed with acetone/methanol (1 : 1) at −20°C for one minute before being stained with 0.5% alcian blue (pH 0.75) overnight at room temperature and washed three times with PBS.

For adipogenic differentiation, the StemPro Adipogenesis Supplement (A10065-01 Gibco, Paisley, UK) was added to the StemPro basal media and the media was changed every 3-4 days over a period of 21 days. The adipogenic cultures were fixed in 4% paraformaldehyde in PIPES buffer for 1 hour, rinsed with DI water, and incubated with 60% isopropanol for 5 minutes at room temperature. Subsequently, the cultures were stained with fresh oil red O solution (three parts 0.3% in isopropanol with two parts water) for 5 minutes and washed three times with PBS.

### 2.6. Migration Experiments

#### 2.6.1. Scratch Assay

MSCs were grown to confluence in a 24-well format and a thin “wound” (800 *μ*m) was introduced by scratching the cell monolayer with a sterile pipette tip. PBMCs were added to the culture (1 : 1) and the open gap was inspected microscopically over time as the cells moved in and filled the damaged area. The migration of the cells from the wound edge into the wound space was recorded using a time-lapse imaging with Eclipse Ti Nikon and analyzed together with Nikon Advanced Research Elements 3.21.00 software.

#### 2.6.2. xCELLigence

The migration and chemoattractant potential of the cells were measured using an xCELLigence system RTCA DP real-time cell analyzer fitted with CIM plates (05665817001, Roche). The CIM plates have 16-well migration chambers comprising upper and lower chambers separated by a porous (pore size 8 *μ*m) polyethylene terephthalate (PET) membrane in conjunction with microelectrodes. The lower chamber of a 16-well CIM plate was filled with cell culture medium, whilst the upper chamber was seeded with 2.0 × 10^4^ cells in medium. In the xCELLigence assay, three test groups were used; (1) MSCs in the upper chamber alone with PBMCs (1 : 1) in the lower chamber to test directed cell movement without cell-to-cell contact, (2) MSCs in the upper chamber alone and 1% FBS as a negative control in the lower chamber, and (3) PBMCs in the upper chamber alone and 1% FBS as a second negative control in the lower chamber. After equilibration, the analyzer was programmed to scan the membrane every 15 minutes for the first 24 hours and thereafter once an hour.

### 2.7. mRNA Expression

Mesenchymal stromal cells were cultured with or without PBMCs (1 : 1) for 24 hours. After the stimulation, the PBMCs were washed away to avoid mRNA from the mononuclear cells grown in suspension. MSC mRNA was extracted using the TRIzol reagent (15596-026, Ambion) according to manufacturer's instructions. The RNA pellet was air-dried and resuspended into 35 *μ*L DNAse/RNAse-free water; subsequently, concentration and quality were checked with OD 260/280 measurement using a NanoDrop spectrophotometer. Quality was verified by agarose 1.2% gel electrophoresis using FlashGel system (57067, Lonza, US) and RNA cassettes (57027, Lonza, US).

#### 2.7.1. PCR Array

cDNA synthesis was performed with RT^2^ first strand kit (330401, Qiagen). For each reaction, 2.8 *μ*g RNA was mixed with 2 *μ*L 5X gDNA elimination buffer, incubated for 5 min at 42°C and chilled on ice. Subsequently, 4 *μ*L 5X Reverse transcription buffer, 2 *μ*L RT enzyme mix reverse transcriptase mix, 1 *μ*L primer and external control mix, and RNAse-free water were added to make a final volume of 10 *μ*L. Reverse transcriptase cocktail and genomic DNA elimination mixture were combined 1 : 1, mixed gently, and incubated for 15 minutes at 42°C, and then immediately the reaction was stopped by heating for 5 minutes at 95°C.

Human mesenchymal stem cell PCR array RT^2^ profiler (PAHS-082Z, Qiagen) and human stem cell RT^2^ profiler PCR array (PAHS-405, Qiagen) were used to identify and compare 84 key genes on each array. A Stratagene Mx3000P real-time cycler was programmed with HotStart DNA Taq Polymerase activation for 10 minutes at 95°C and then 40 cycles of (1) denaturation for 15 s at 95°C and (2) combined annealing/extension for 1 minute at 60°C. The data acquisition was performed during the combined annealing/extension step and the results were analyzed with RT^2^ Profiler PCR array data analysis software version 3.5 using the ΔΔCt method and normalized to the mean of five housekeeping genes used (B2M, HPRT1, RPL13A, GAPDH, and ACTB).

#### 2.7.2. Quantitative rtPCR

The cDNA synthesis was performed with SuperScript VILO kit (11754-050, Invitrogen). For each reaction, up to 2.5 *μ*g RNA was mixed with 4 *μ*L VILO reaction mix, 2 *μ*L 10X SuperScript enzyme, and DEPC-treated water added until to a total of 20 *μ*L. The reaction mixture was incubated for 10 minutes at 25°C and then 60 minutes at 42°C and finally the reaction was terminated with 5 minutes at 85°C.

The real-time quantitative PCR reaction was prepared using QuantiFast SYBR Green (204054, Qiagen). A total volume of 25 *μ*L of reaction was prepared with 12.5 *μ*L 2x QuantiFast SYBR Green PCR Master Mix, 2.5 *μ*L QuantiTect Primer Assay (Hs_BMP2_1_SG, Hs_BMP6_1_SG, Hs_GDF5_1_SG, Hs_GDF6_1_SG, and Hs_SOX9_1_SG), 50 ng template cDNA, and RNase-free water. Stratagene Mx3000P real-time cycler was programmed with an initial heat activation for 5 min at 95°C followed by a 2-step cycling; firstly denaturation for 10 s at 95°C, followed by a combined annealing/extension for 30 s at 60°C, repeated for 40 cycles. The data acquisition was performed during the combined annealing/extension step. The relative copy numbers of target genes were calculated from the standard curve for each gene and normalized to the housekeeping gene beta-2 microglobulin (B2M).

### 2.8. Data Analysis

All samples were collected in four replicates and the data is presented as the mean ± standard deviation (SD). The data were evaluated using Student's *t*-test to determine statistically significant differences with GraphPad Prism 5 software package. All data were confirmed for normal distribution with the significance level set at 0.05.

## 3. Results

### 3.1. MSC Characterization

**Figure 1:**
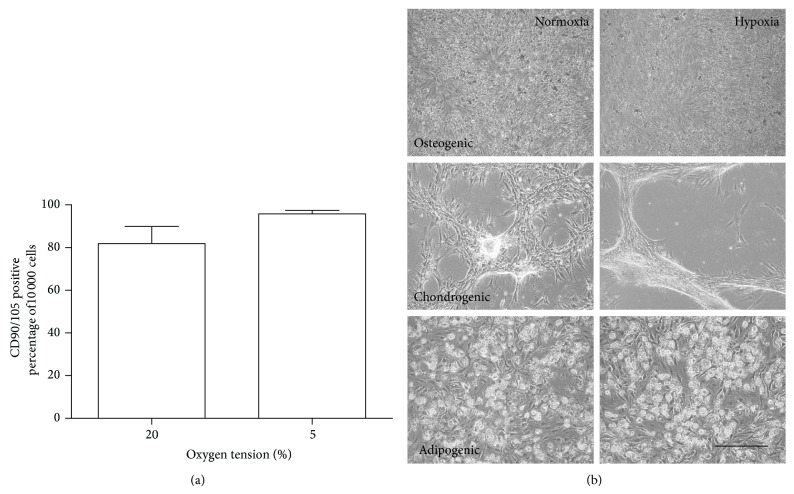
(a) Flow cytometry result of CD90 and CD105 expression in human primary MSCs (*n* = 6) both normoxia and hypoxia. Results are expressed as mean % of positive cells in the whole population. (b) Multidifferentiation assay for osteogenic (alizarin red), chondrogenic (alcian blue), and adipogenic (oil red O) staining of MSC cultures in both normoxia and hypoxia at day 21 (scale bar 200 nm, *n* = 4).

Adipose tissue-derived MSCs isolated from the infrapatellar fat pad presented CD90 and CD105 cell surface markers typical of an MSC phenotype lacking the CD34/45 expression (measured at passage 4). Hypoxic culture environment similar to that of native cartilage tissue did not have significant effect on MSC progenitor cell population (95.7 ± 3.3% CD90/CD105 positive) when compared to the normoxic culture (84.1 ± 17.0% CD90/CD105 positive) (*P* = 0.07, Figure 1(a)) although there was a beneficial trend.

Staining of MSCs after 21 days in differentiation media showed that the resultant cells displayed phenotypic characteristics of osteogenic, chondrogenic, and adipogenic pathways* in vitro* (Figure 1(b)). Osteogenic differentiation of cultured cells formed mineralizing aggregates that stained with an alizarin red. The characteristic accumulation of matrix in chondrogenesis was observed with an alcian blue staining of developing chondrogenic pellets. Adipogenesis was indicated by the accumulation of neutral lipid vacuoles that stained with an oil red O staining. Low oxygen tension was not found to reduce the differentiation potential of MSCs. Therefore, the functional differentiation assay demonstrated that the multipotent differentiation capability of MSCs was independent of oxygen tension.

### 3.2. Evaluating MSC/PBMC Coculture

#### 3.2.1. Cell Migration

**Figure 2:**
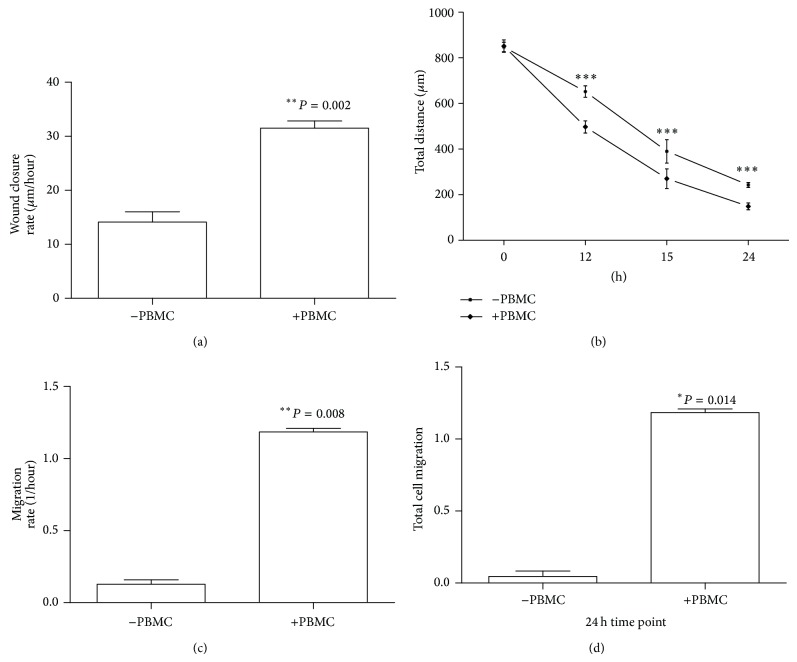
Cell migration experiment of MSCs measured over 24 hours comparing the effect of PBMC stimulus (*n* = 4). (a) Wound closure rate measured at 3-hour time point (*n* = 4) in the scratch assay with direct cell-to-cell contact. (b) The total distance of the wound measured at 12-, 15-, and 24-hour time points is not to scale (*n* = 4, *P* < 0.0001) in the scratch assay. (c) The initial cell migration rate at 3-hour time point quantified real time with the xCELLigence system and (d) the total amount of cells migrated at the 24-hour time point quantified with cell index value by xCELLigence.

Scratch assay showed that direct cell-to-cell PBMC stimulation increased the wound closure rate by 55.2% at 3 hours, *P* = 0.002 (Figure 2(a)). The distance of the gap was measured at 12-, 15-, and 24-hour time points (Figure 2(b)) and every time point the PBMC treated wound had smaller scratch area (*P* < 0.0001). To confirm the findings from the scratch assay and to assess if direct cell-to-cell contact is needed for the chemotactic effect, a second assay based on the Boyden chamber model (xCELLigence) was used to quantify cell migration. The real-time cell migration assay demonstrated that during the first 3 hours the MSC migration was 9 times faster in the PBMC stimulated test group (*P* = 0.008, Figure 2(c)). The results of the 24-hour migration assay show that* in vitro* MSC migration was 25 times higher when stimulated with the PBMCs (*P* = 0.014, Figure 2(d)) even though the cells did not have direct cell-to-cell contact. The directed migration was not found in the negative controls.

#### 3.2.2. mRNA Expression

**Figure 3:**
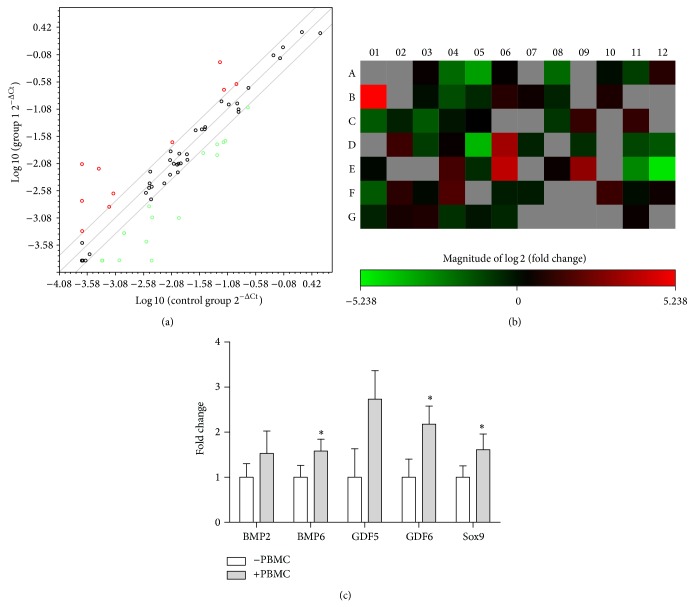
Change in mRNA levels after 24 h PBMC stimulation with a cut-off value of 4. (a) A scatter plot showing up- and downregulated genes and core genes with no change (*n* = 1). (b) A heat map visualization of 2log2-fold change of the 84 genes in the stem cell PCR array (red: upregulated and green: downregulated). Grey shows the genes that were undetermined (no Ct value with a cut-off value of 35). (c) Real-time PCR validation of 5 key chondrogenic genes (*n* = 4) normalized to B2M housekeeping gene mRNA levels.

In total the expressions of 158 genes related to stem cell differentiation, growth, pluripotency, self-renewal, and the mesenchymal lineage commitment pathways were measured using a commercial array. In total mRNA levels of 52 genes involved in MSC differentiation and growth were upregulated by the PBMC stimulation as compared to the unstimulated test group. Stemness marker mRNA levels for FGF2, INS, LIF, SOX2, TERT, WNT3A, and ZFP42 were upregulated (>15-fold) by PBMC stimulation. The mRNA levels of 19 genes specific to MSCs (ALCAM, ANPEP, BMP2, CASP3, CD44, ENG, ERBB2 (HER2), FUT4, FZD9, ITGA6, ITGAV, KDR, MCAM, NGFR, NT5E, PDGFRB, PROM1, THY1, and VCAM1) were also upregulated in PBMC stimulated MSCs. A partial effect was seen on the mRNA levels of 11 genes specific to osteogenesis, where 7 of them (BMP2, BMP6, FGF10, HNF1A, KDR, RUNX2, and TBX5) were upregulated by 24-hour PBMC stimulation. Messenger-RNA levels for 8 genes coding ABCB1, BMP2, BMP6, GDF5, GDF6, GDF7, ITGAX, and SOX9 proteins specific to chondrogenic differentiation were upregulated by over 15-fold following PBMC stimulation. Real-time PCR was used to further investigate five key chondrogenic genes and the results confirm that mRNA levels for all these genes were upregulated by the PBMCs (Figure 3(c): BMP2, BMP6 (*P* = 0.049), GDF5, GDF6 (*P* = 0.028), and Sox9 (*P* = 0.043)).

## 4. Conclusions

A major issue in cartilage repair is the lack of chondrogenic cells migrating from healthy tissue into damaged areas whilst maintaining their phenotype. In addition, unlike the majority of tissues, cartilage healing does not involve mononuclear cells as it is essentially avascular. To our knowledge, no previous publications have reported the use of PBMCs as a source of chemoattractants in cartilage tissue repair. Our aims in this study were to establish whether PBMCs had a positive influence on MSC migration and phenotype.

In both scratch assay and real-time Boyden chamber analysis, PBMCs were found to stimulate MSC migration. The addition of PBMCs was found to increase the MSC cell migration rate by 55% at the 3-hour time point (*P* = 0.002) in a scratch assay with direct cell-to-cell contact. The Boyden chamber real-time cell migration assay confirmed that during the first 3 hours the MSC migration rate was 9 times faster in the PBMC stimulated test group (*P* = 0.008) compared to nonstimulated one. The results of the 36-hour migration assay show that* in vitro* MSC migration rose to 25 times higher when stimulated with the PBMCs (*P* = 0.014). These results clearly demonstrate that PBMCs can induce MSC motility and increase both the total number of cell migration as well as the rate of cell movement. Additionally, the xCELLigence assay showed that no direct cell-to-cell contact was required as the directed cell movement was observed when MSCs were in the upper chamber separated from the PBMCs in the lower chamber. The migration of MSCs is known to be regulated by a variety of cytokines, such as FGF-2, PDGF, and MIF [42–45]. Among the PBMC released cytokines, one possible candidate for future study is monocyte chemoattractant protein 1 (MCP-1) as it has been shown to mediate migration of MSCs in a rat aortic-allograft model [46]. Future work should investigate the individual chemotactic factor or factors present in heterogeneous PBMCs in order to harness the full potential of the directed cell migration property.

Physiologically the infrapatellar fat pad is found in close vicinity to articular cartilage in the knee making it a prospective tissue type to recruit stem cells for tissue repair [19]. In our study, MSCs derived from the infrapatellar fat pad at passage three were over 84% positive for the MSC markers and this could be even further induced to over 96% by using hypoxic cell culture conditions. This is an important finding and warrants future studies to evaluate the biological mechanism by which hypoxia appears to either support or select (selective cell survival) the progenitor cell phenotype.

The multidifferentiation potential of infrapatellar fat pad derived progenitor cells was confirmed with a functional assay showing that when cultured in an appropriate cell culture environment, the cells displayed many of the phenotypic characteristics of cells of the osteogenic, chondrogenic, and adipogenic lineages. Unlike previous studies [47, 48], hypoxic culture conditions did not improve the chondrogenic differentiation capability of MSCs in this current study. Successful hypoxic preconditioning may be related to the timing of hypoxia as this appears to have an important function early in the lineage progression, modulating colony formation and proliferation. The fact that hypoxia did not reduce the differentiation potential of MSCs in our model is encouraging and allows infrapatellar fat pad derived MSCs to be considered as a viable candidate to become a suitable cell source in therapeutic cartilage repair. Further research is needed to elucidate the biological effect of low oxygen tension on infrapatellar fat pad-derived progenitor cell types.

Since no studies have reported the use of MSCs together with PBMCs, this study serves as the original basis to evaluate this coculture model. The human stem cell and mesenchymal stem cell PCR array results showed that human MSCs derived from infrapatellar fat pad expressed key markers for cell cycle regulators, chromosome and chromatin modulators, genes regulating symmetric/asymmetric cell division, self-renewal markers, and cell adhesion molecules as well as signaling pathways important for stem cell maintenance including the Notch- and Wnt-pathways. MSCs expressed mesenchymal lineage markers but lacked the expression for embryonic and hematopoietic cell lineage markers (Ct > 35, data not shown). The 24-hour PBMC stimulation produced an increase in mRNA levels for 52 genes related to mesenchymal lineage commitment. Seven genes of these are known to be key in control of the stemness of the mesenchymal stem cells (FGF2, INS, LIF, SOX2, TERT, WNT3A, and ZFP42).

The ability of adipose-derived stem cells to undergo chondrogenic differentiation has been studied extensively, and it has been suggested that the most promising growth factors for chondrogenesis appear to be TGFbeta-3 and bone morphogenetic protein- (BMP-) 6 [49], both of which gene transcripts were upregulated by the PBMC stimulation on our study. The MSC specific PCR array in this paper confirms the presence of MSC-specific markers and provides evidence that infrapatellar fat pad derived stem cells can express differentiation markers for osteogenic and chondrogenic lineages and, moreover, PBMCs can further stimulate the expression of differentiation markers. Eight genes associated with chondrogenic differentiation (ABCB1, BMP2, BMP6, GDF5, GDF6, GDF7, ITGAX, and SOX9) were upregulated by over 15-fold by the PBMC stimulation. This positive finding was confirmed using qPCR with five key chondrogenic genes (BMP2, BMP6 (*P* = 0.049), GDF5, GDF6 (*P* = 0.028), and Sox9 (*P* = 0.043)) demonstrating that PBMCs have a positive reparative influence on MSCs in cartilage.

Taken together, this study presents a readily available source of cells from the peripheral blood to support migration and chondrogenic differentiation of mesenchymal stromal cells from the infrapatellar fat pad in the knee. The ability to considerably enhance regeneration of functional cartilage from adult human mesenchymal stromal cells would have tremendous clinical impact. Other studies are currently under way using a large animal model to examine the therapeutic benefits of these cells to repair articular surface and osteochondral defects.
